# Novel Allogeneic Mitochondria and Associated Organelle Complex Treatment Prevents Myocardial Ischemia-Reperfusion Injury Through Anti-Apoptotic Effects

**DOI:** 10.1016/j.jacasi.2025.09.022

**Published:** 2025-11-18

**Authors:** Takumi Hayashi, Yuuki Shimizu, Hisashi Ota, Keiichi Sakakibara, Haihang Luo, Takahiro Shibata, Yiyang Che, Tianyu Liu, Rei Shibata, Rick Tsai, Masashi Suganuma, Toyoaki Murohara

**Affiliations:** a Department of Cardiology, Nagoya University Graduate School of Medicine, Nagoya, Japan; b Department of Advanced Cardiovascular Therapeutics, Nagoya University Graduate School of Medicine, Nagoya, Japan; c LUCA Science, Inc, Tokyo, Japan

**Keywords:** anti-apoptosis, cardioprotection, cryopreservation, ischemic reperfusion injury, mitochondria

## Abstract

**Background:**

Ischemia-reperfusion (I/R) injury can damage mitochondria and lead to cardiomyocyte apoptosis. Freshly prepared autologous mitochondria have shown benefits against I/R injury, but it remains unclear whether allogeneic mitochondrial organelle complex Q (MRC-Q) after freeze-thawing can also be effective in this context.

**Objectives:**

This study aimed to determine whether administering MRC-Q can protect the heart from ischemia I/R injury in animal models.

**Methods:**

The MRC-Q used in our current experiments was intactly isolated by proprietary technology and cryopreserved. A mouse and porcine model of cardiac I/R injury was employed. In vitro experiments were performed using H9C2 cardiomyocytes subjected to hypoxic-reoxygenation conditions.

**Results:**

Our initial study demonstrated that MRC-Q can be storable with a maintenance of adenosine triphosphate production capacity. Next, we tested cardioprotective effects of MRC-Q administration on I/R injury in a murine model. Compared to the vehicle group, the MRC-Q–treated group showed a reduction in infarct size with fewer apoptotic cells, a decrease in circulating cardiac enzymes, and an improvement in cardiac function. In vitro studies revealed that MRC-Q was taken up into cardiomyocyte cells in a time-dependent manner. Next, we demonstrated that cell viability was improved in the MRC-Q–treated group after hypoxia/reoxygenation. Further examinations suggested that one of the mechanisms of cardioprotection by MRC-Q could be mediated by the secretion of mitochondria-derived peptides, which could inhibit apoptosis-promoting signals. In addition, MRC-Q augmented endogenous mitochondrial quality control in injured myocytes followed by up-regulation of adenosine triphosphate production and anti–reactive oxygen species activity. Finally, a preclinical pig model also confirmed the cardioprotective effects of MRC-Q administration.

**Conclusions:**

Our data demonstrated that freeze-thawed MRC-Q have a protective effect on the heart after I/R injury.

Although substantial progress has been made in the diagnosis and treatment of acute coronary syndromes, cardiovascular disease remains the leading cause of death globally, with nearly one-half of these deaths due to ischemic heart disease.^1^ For instance, the long-term prognosis of acute coronary syndromes has recently been improved by percutaneous coronary intervention reperfusion therapy, the prognosis is markedly worse when reperfusion injury develops as a complication of this therapy.^2^ Despite the combination of treatment strategies, including the use of drugs to prevent reperfusion injury such as nicorandil, preconditioning interventions, and peripheral embolic protection devices, having shown some efficacy, it remains a major clinical challenge.^3^^,^^4^

Mitochondria are essential organelles found in most cell types excluding red blood cells, which are responsible for producing energy in the form of adenosine triphosphate (ATP) through a process called oxidative phosphorylation.^5^ They are also key regulators of cell death and survival, playing a crucial role in the development of reperfusion injury.^5^ For instance, during periods of ischemia, the lack of oxygen and nutrients can lead to mitochondrial dysfunction, which can impair energy production and increase the production of reactive oxygen species (ROS), leading to oxidative stress and damage to the mitochondria and other cell structures.^6^^,^^7^ This mitochondrial dysfunction and damage can contribute to the development of reperfusion injury and worsen tissue damage and inflammation.^8^^,^^9^ Therefore, strategies to protect and enhance mitochondrial function and/or number during ischemia and reperfusion may help reduce the severity of reperfusion injury and improve outcomes.^7^^,^^10^, ^11^, ^12^ These strategies include using mitochondria-targeted antioxidants and other agents that can enhance mitochondrial function and reduce ROS production.^11^^,^^13^ Despite these vigorous efforts, there are still few treatments for mitochondrial dysfunction in actual clinical practice.^14^ However, recent evidence has demonstrated that mitochondria transfer between cells improves cellular functions in pathological or energy-deficit conditions.^15^, ^16^, ^17^ The damaged cells receive mitochondria from healthy cells, contributing to cytoprotective provided by self-endogenous.^18^^,^^19^ A novel therapeutic approach has been proposed in which autologous mitochondria are isolated and freshly transplanted into damaged tissues to provide organ protection.^20^, ^21^, ^22^, ^23^

More recently, it has been reported that the mitochondria named mitochondrial organelle complex Q (MRC-Q) can be extracted and stored as biologics through several of innovative technologies (WO2021132735A2).^24^ However, it is not known whether exogenously administered freeze-thawed MRC-Q can be replenished in the heart to achieve cardioprotective effects after ischemia-reperfusion (I/R) injury. If any, the detailed therapeutic mechanism has not been fully elucidated.

Accordingly, the aim of this study was to investigate whether the administration of freeze-thawed mitochondria leads to a protective effect on the myocardium after I/R injury using a well-established murine model and a swine preclinical model. Furthermore, we explored its potential cytoprotective mechanism.

## Methods

The data, analytic methods, and materials employed in this study are available from the corresponding author on reasonable request.

### Animal studies

All procedures for animal care and study were approved by the Animal Ethics Review Board of the Nagoya University School of Medicine. Our study conformed to the guidelines from the National Institute of Health Guide for the Care and Use of Laboratory Animals. C57BL6J mice were purchased from Charles River Laboratories Japan Inc and were randomly assigned to experimental groups. We identified all mice with each ear tag throughout the series of studies to ensure blindness. Before the surgical procedure, mice were anesthetized with a combination of hydrochloric acid medetomidine (0.3 mg/kg), midazolam (4 mg/kg), and butorphanol tartrate (5 mg/kg) intraperitoneally.^25^ Cervical dislocation under anesthesia was used for euthanasia.^25^

### Mitochondria isolation

The mitochondria used in the experiments in this study (allogeneic MRC-Q) were purified by the intact Mitochondria Isolation Technology (iMIT) without homogenization/solubilization by the LUCA Science Inc. Briefly, cells are incubated with low concentration of detergent without making pores in cell. Gentle pipetting results in distraction of cell membrane and extraction of mitochondria (WO2021132735A2).^24^^,^^26^ All MRC-Q was frozen and stored at −80 °C before use.

### Mouse myocardial I/R injury model

Mice were cannulated the trachea with a polyethylene tube connected to a respirator with a tidal volume of 0.6 mL (200 breaths/min) after the sedations.

Chest hair was removed, and mice were placed on their back on a heated table (37 °C). We performed thoracotomy and ligated left coronary artery under a microscope with 8-0 ETHILON suture using PE-10 tube.^27^ The ST-segment elevation of the electrocardiograph was noted after left anterior descending (LAD) artery ligation (Supplemental Figure 1A), and the apical part of the heart was blanched consistent with tissue ischemia. After ligation for 45 or 60 minutes, the polyethylene-10 tube was released to induce reperfusion and 10 μg of MRC-Q resuspended in 10 μL of phosphate-buffered saline (PBS) were injected into the 6 points (1-2 μL of each, total 10 μL per animal) by micro syringe (#DS80901, model 1705 LT Syringe, Hamilton) and micro needle (#90134, 30-gauge, Kel-F Hub Needle, Hamilton) at the damaged myocardium of the left ventricle (LV).^10^^,^^11^^,^^28^ For placebo-treated I/R mice, 10 μL PBS were injected intramyocardially into different 6 points at the damaged myocardium of the LV.

### Cardiac function assessment

Twenty-four hours after operation, systolic cardiac functional parameters were assessed (Vevo 1100 imaging system, FUJIFILM Visual Sonics, Inc). LV end-systolic diameter, LV end-systolic volume, LV end-diastolic diameter, LV end-diastolic volume, fractional shortening, and ejection fraction were calculated from M-mode images.^29^

### Evans blue and triphenyltetrazolium chloride staining

After 24 hours of reperfusion, mice were anesthetized. We retied the suture and injected 1 mL of 1.0% Evans blue to determine the nonischemic area, then excised the heart, washed it with PBS, and cut it into thin slices (short-axis slices).^11^ All groups were evaluated using the fourth slice from the apex, obtained by dividing the heart into 5 equal segments. These slices correspond to the papillary muscle level, which we consider appropriate for assessing the infarcted region in this model.^11^^,^^29^, ^30^, ^31^ We stained slices with 2,3,5-triphenyltetrazolium chloride to determine infarct area and photographed them under a microscope. LV area, area at risk (AAR), and infarct area were determined by Image J software (National Institutes of Health).^11^

### Pig model of I/R injury

This study used domestic female swine (body weight 35-40 kg). The I/R- procedure was performed as previously described.^31^ Briefly, after animals were anesthetized, coronary angiography was performed to determine the optimal location of the occlusion. After an over-the-wire-type angioplasty balloon catheter was placed in the LAD artery at the first major diagonal branch, the balloon was inflated to occlude the LAD artery. An intracoronary bolus of MRC-Q (250 μg per animal)^21^^,^^32^ or vehicle (saline) as a control was given through the wire lumen of the inflated balloon catheter just before the time of reperfusion after 90 minutes’ occlusion of the LAD artery.^31^

### Cell culture

H9c2 cells were originally derived from embryonic rat ventricular cardiomyocytes and purchased from ATCC.^33^ Cells were grown in Dulbecco modified Eagle medium (Sigma) supplemented with 10% fetal bovine serum and 1% penicillin/streptomycin. All cells were maintained in a humidified culture incubator under 5% CO_2_ at 37 °C. After H9c2 cells reached 70% confluence, to simulate myocardial I/R injury in vivo, hypoxia/reoxygenation (H/R) was performed on H9c2 cells.^34^ H9c2 cells were exposed to hypoxia for 12 hours in a hypoxic incubator (94% N_2_, 5% CO_2_, and 1% O_2_ at 37 °C).^31^ After hypoxic exposure, the cells were reoxygenated for up to 24 hours at 37 °C in a normoxic incubator.^31^

### Isolation of RNA and real-time reverse transcription PCR

Total RNA was isolated using the RNeasy Mini Kit (QIAGEN) from mouse cardiac tissue.^35^ Reverse transcription was performed using a quantitative polymerase chain reaction (PCR) RT master mix kit (TOYOBO).^28^^,^^35^ Real-time reverse transcription–PCR analysis was performed on a C1000 Thermal Cycler (BIO-RAD) using SYBR Green I and the following conditions: 95 °C for 10 minutes followed by 40 cycles at 95 °C for 15 seconds and 60 °C for 45 seconds.^35^ The expression of target messenger RNAs was normalized to that of GAPDH in each sample. Primer sequences are described in the Supplemental Appendix.

### Enzyme-linked immunosorbent assay

Abdominal vein blood samples were centrifuged at 3,000*g*. Then, the serum samples were harvested. Following the instructions of enzyme-linked immunosorbent assay kits, B-type natriuretic peptide, creative kinase isoenzyme MB, and cardiac troponin I levels were tested in serum or cell supernatant.^36^ Through automatic biochemical analyzer, lactate dehydrogenase and cardiac troponin I levels were examined in serum or cell supernatant.

### Histology and immunohistochemistry

Heart samples from each group were embedded in an optimal cutting temperature compound (Sakura). Frozen sections (5-μm thickness) were used for histological analysis. Heart tissue fibrosis was determined by Masson trichrome staining, following the manufacturer’s protocol (Sigma).^29^ Briefly, heart sections were sequentially incubated in the following solutions. Bouin solution (overnight), Weigert iron hematoxylin working solution (5 minutes), Biebrich scarlet acid solution (2 minutes), phosphomolybdic-phosphotungstic acid solution (5 minutes), aniline blue solution (10 minutes), and 1% acetic acid (2 minutes).^29^ Images were visualized on a BZ-X710 fluorescence microscope (KEYENCE).

### Electron microscopy

H9c2 cells were fixed with 2.5% glutaraldehyde in 0.1 mol/L sodium phosphate buffer, pH7.4 and the post-fixed with 2% osmium tetraoxide for 1 hour. Cells were then dehydrated with a series of graded ethanol and finally embedded in Epon812 resin for 48 hours. Images were acquired in transmission electron microscopy JEM-1400 Flash (JEOL Ltd) at 100 kV. The images were captured from comparable regions for comparative analysis.

### DHE staining

Dihydroethidium (DHE) staining was performed according to the manufacturer’s protocols.^11^ The 5-μm-thick frozen heart sections were first incubated with DHE solution at 37 °C for 30 minutes. Staining images were visualized on a BZ-X710 fluorescence microscope.^37^

### TUNEL assay

Terminal uridine nick-end labeling (TUNEL) assay was performed to evaluate DNA damage and fragmentation in cell apoptosis.^27^ H9c2 cells were washed twice with PBS. Following fixation with 4% paraformaldehyde for 15 minutes at room temperature, cells were incubated with 15 μg/mL proteinase K for 15 minutes at 37 °C and endogenous peroxidase activity was blocked using 3% H_2_O_2_ for 15 minutes at room temperature.^27^ After washing, cells were treated with TUNEL working solution for 60 minutes at 37 °C, and cells were captured using BZ-X710 fluorescence microscope.^27^

### Necrotic assay

Necrosis was measured by the Necrotic Assay Kit (#ab176749, Abcam).

### Caspase detection

Caspase-3/7 was measured by the Apo-ONE Homogeneous Caspase-3/7 Assay kit (#G7792, Promega).

### Western blots

Samples collected from isolated mitochondria, experimental whole heart were homogenized, and lysates were prepared for Western blot experiments.^38^ Western blot analysis was performed as previously described, using antibodies against TFAM (#8076, 1:1,000; Cell Signaling Technology [CST]), TOMM20 (#sc-17764, 1:1,000, Santa Cruz Biotechnology), citrate synthase (#14309, 1:1,000, CST), Complex Ⅰ (#45-8199, OxPhos Human WB Antibody Cocktail, 1:10,000, Thermo Fisher Scientific), Complex Ⅱ (#45-8199, OxPhos Human WB Antibody Cocktail, 1:1,000, Thermo Fisher Scientific), Complex Ⅲ (#45-8199, OxPhos Human WB Antibody Cocktail, 1:1,000, Thermo Fisher Scientific), Complex Ⅳ (#45-8199, OxPhos Human WB Antibody Cocktail, 1:1,000, Thermo Fisher Scientific), calreticulin (#12238, 1:1,000, CST), golgin97 (#13192, 1:1,000, CST), catalase (#12980, 1:1,000, CST), P70s6k (#2708, 1:1,000, CST), Bax (#2772S, 1:1,000, CST), apoptosis-inducing factor (#4642S, 1:1,000, CST), caspase 3 (#9662, 1:1,000, CST), cleaved caspase 3 (#9661S, 1:1,000, CST), GAPDH (#2118S, 1:1,000, CST).^38^

### Statistical analysis

For biometrical sample size estimation, G∗Power (version 3.1.3) was used.^39^ Calculation of appropriate sample size groups was performed using a priori power analyses by comparing the mean of 2-3 groups with a defined adequate power of 0.8 (1 − β error) and an α error of 0.05. To determine the prespecified effect size *d* or *f*, previously published data were considered as comparable reference values.^11^^,^^29^^,^^31^^,^^32^ The number of individual animals per group (number of biologically independent samples) for each experiment and the meaning of each data point are indicated in the respective figure legends. Shapiro-Wilk normality test was performed to evaluate data distribution. Normally distributed data with 1 variable were analyzed by the unpaired Student’s *t*-test to evaluate the statistical significance between the 2 groups. One-way analysis of variance, along with Tukey post hoc test, was used for 3 or more groups. We also used a 2-way repeated-measures analysis of variance (Sidak post hoc tests) to assess the changes over time. Non-normally distributed data were analyzed by 2-tailed Mann-Whitney *U* test between 2 groups. Continuous parametric data were expressed as mean ± SEM, and in the case of nonparametric data, the median (IQR) were reported. GraphPad Prism software (version 8.0, GraphPad Software Inc) was used for the statistical analysis. Statistical significance was defined as *P* < 0.05.^40^

## Results

### Integrity and characterization of MRC-Q

**Figure 1:**
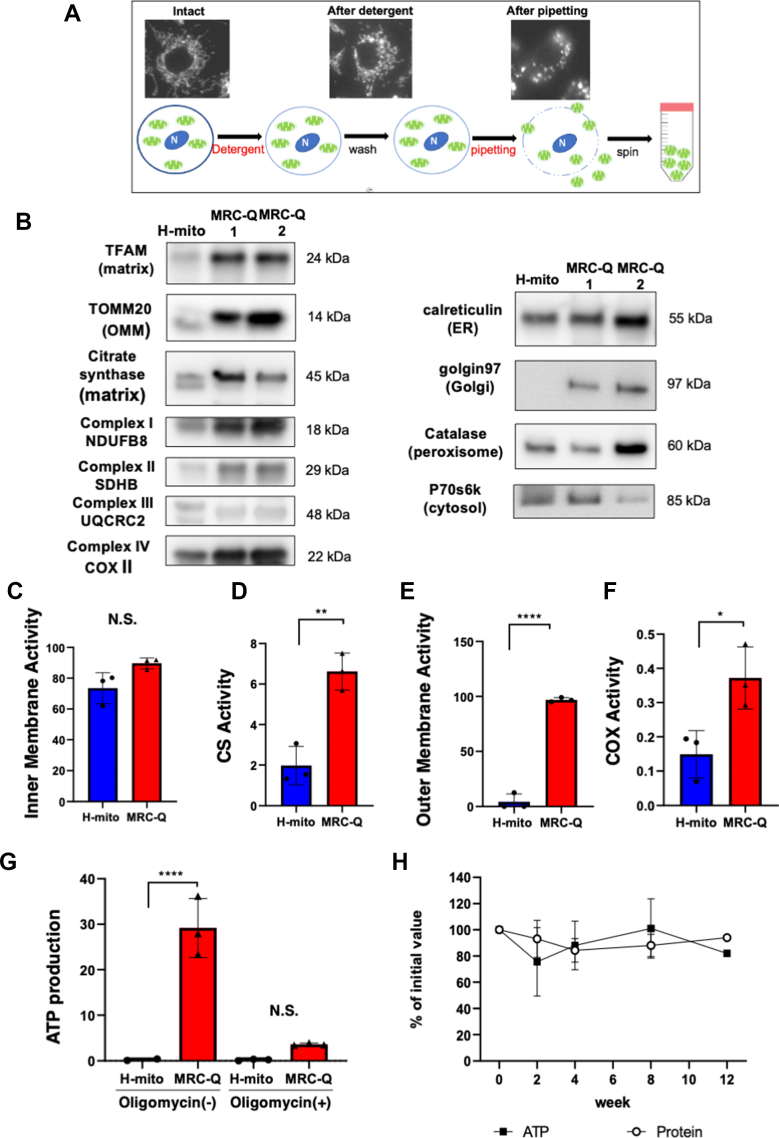
Character of MRC-Q (A) A simplified scheme of the protocol for the intact Mitochondria Isolation Technology (iMIT). (B) Western blotting on mitochondria with different isolation methods. A consistent protein quantity of 10 μg per lane was loaded. Representative photographs by Western blot in homogenized mitochondria (H-mito), mitochondrial organelle complex Q (MRC-Q) with iMIT at room temperature (3 minutes), MRC-Q with iMIT at 4 °C (3 minutes). (C,D) Inner membrane integrity was calculated by measurement of 5-thio-2-nitrobenzoic acid formed from coenzyme A-SH and DTNB. Coenzyme A-SH was a byproduct of citrate from oxaloacetic acid and acetyl coenzyme A by citrate synthetase (CS). Membrane integrity (%) = (amount of end-product with detergent – amount of end-product without detergent)/amount of end-product with detergent. These were normalized by total mitochondrial protein amount. Results are presented as mean ± SEM (n = 3). ∗∗*P* = 0.003 vs H-mito. (E,F) Outer membrane integrity was calculated by measurement of oxidized cytochrome c from reduced cytochrome c by cytochrome c oxidase (COX). Membrane integrity (%) = (amount of end-product with detergent – amount of end-product without detergent)/amount of end-product with detergent. These were normalized by total mitochondrial protein amount. Results are presented as mean ± SEM (*n* = 3). ∗∗∗∗*P* < 0.0001 vs H-mito, ∗*P* = 0.027 vs H-mito. (G) Adenosine triphosphate (ATP) was measured by CellTiter-Glo kit (Promega) with MRC-Q and H-mito (0.5 mg protein) with/without oligomycin (5 mmol/L). Results are presented as mean ± SEM (*n* = 3). ∗∗∗∗*P* < 0.0001 vs H-mito. (H) Percentage of initial value of ATP and protein after storage in liquid N_2_. Results are presented as mean ± SEM (n = 3). ER = endoplasmic reticulum; NS = not significant; OMM = outer mitochondrial membrane.

The mitochondria used in the current experiments were isolated by a proprietary technology (WO2021132735A2) (MRC-Q) from human umbilical vein endothelial cells (Figure 1A). Our initial experiment demonstrated that MRC-Q isolated by the novel technique was found to retain mitochondrial components compared to mitochondria isolated by conventional homogenization methods (Figure 1B). Initially, MRC-Q was developed by separation at room temperature, as was the homogenized control method. Subsequently, to obtain a higher quality formulation, a comparison was made between the iMIT method prepared at 4 °C and room temperature conditions (Figure 1B). As a result of Figure 1B, subsequent studies were conducted using the iMIT method prepared at 4 °C. In addition, mitochondrial function of MRC-Q in terms of citrate synthetase activity, outer membrane activity, and cytochrome c oxidase activity were superior to that of conventionally isolated mitochondria (Figures 1C to 1F). As a results, ATP production capacity in MRC-Q was greater than that of homogenized mitochondria (Figure 1G). Importantly, MRC-Q was found to be storable, with preserved ATP production capacity and the protein recovery rate more than 80% of the initial value (ie, baseline) at storage for at least 12 weeks (Figure 1H).

### Treatment with MRC-Q reduces myocardial infarct size suppressing cardiomyocyte apoptosis and inflammatory responses after I/R in a murine model

Next, we tested whether MRC-Q could have beneficial effects on acute myocardial I/R injury. Male C57BL/6J mice (Charles River Laboratories Japan Inc, Kanagawa, Japan) were subjected to 45 minutes of myocardial ischemia, then intramuscularly injected with or without MRC-Q, followed by 24 hours of reperfusion.

**Figure 2:**
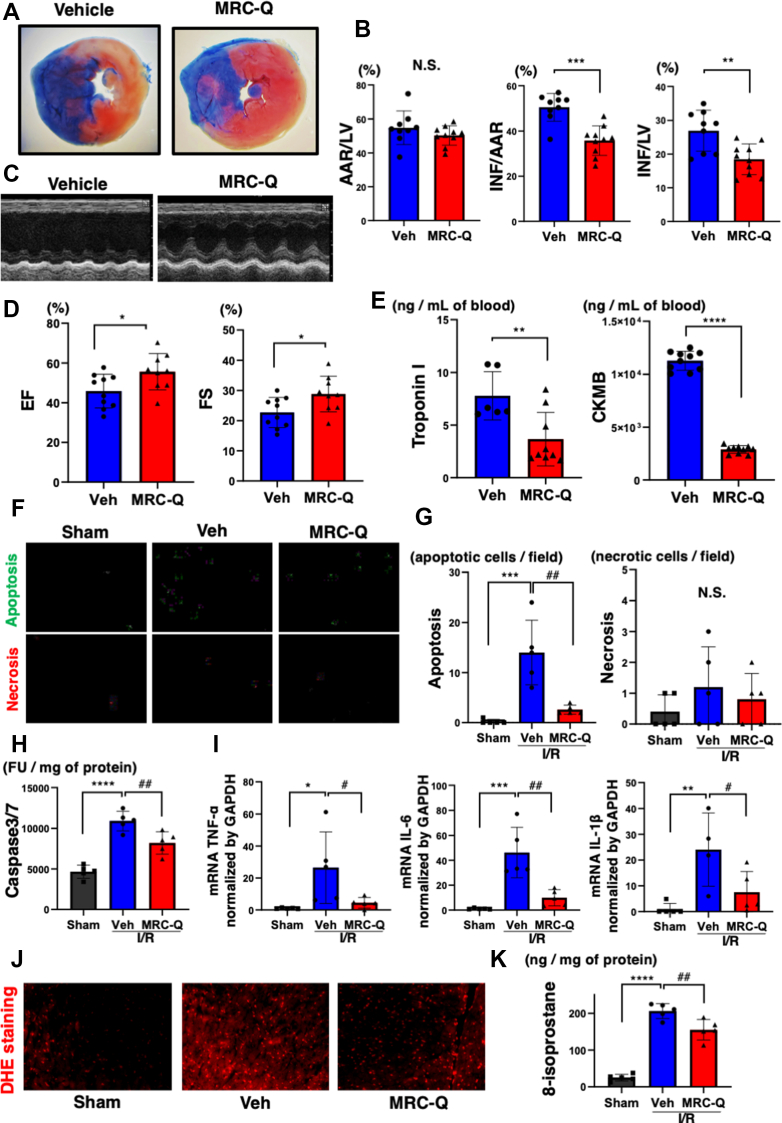
MRC-Q Attenuated Infarct Size and Improved Cardiac Function After I/R Injury (A) Representative examples of myocardial infarction stained with Evans blue and triphenyl tetrazolium chloride 24 hours after reperfusion. Blue area depicts perfused tissue; red plus white, at-risk tissue; white, infarcted tissue. (B) Bar graphs depict the area at risk (AAR) as a percentage of the left ventricular (LV) area, infarct size as a percentage of the AAR, and infarct size as a percentage of the LV area. Results are presented as mean ± SEM from vehicle (Veh) (n = 9) and MRC-Q (n = 10). ∗∗∗*P* = 0.001 vs Veh, ∗∗*P* = 0.003 vs Veh. (C,D) Ejection fraction (EF) and fractional shortening (FS) percentages were examined by echocardiography (M-mode). Results are presented as mean ± SEM from Veh (n = 10) and MRC-Q (n = 9). ∗*P* = 0.027 vs Veh (EF%), ∗*P* = 0.025 vs Veh (FS%). (E) Plasma troponin I and creative kinase isoenzyme MB (CK-MB) concentration. Results are presented as mean ± SEM from Veh (n = 6) and MRC-Q (n = 9). ∗∗*P* = 0.007 vs Veh, ∗∗∗∗*P* < 0.0001 vs Veh. (F) Results from immunohistochemistry of heart tissue sections showing apoptotic cardiomyocytes in green fluorescence and necrotic cardiomyocytes in red fluorescence. (G) Apoptotic and necrotic cardiomyocytes expressed as the percentage of total myocytes. Results are presented as mean ± SEM (n = 5). ∗∗∗*P* = 0.0002 vs sham, ##*P* = 0.0012 vs Veh, by 1-way analysis of variance (ANOVA) and Tukey post hoc tests. (H) The activity of caspase 3/7. Results are presented as mean ± SEM (n = 5). ∗∗∗∗*P* < 0.0001 vs sham, ##*P* = 0.008 vs Veh, by 1-way ANOVA and Tukey post hoc tests. (I) The messenger RNA (mRNA) expression levels of TNF-α, IL6, and IL1β. Results are presented as mean ± SEM (n = 5). ∗*P* = 0.023 vs sham, #*P* = 0.049 vs Veh (TNF-α), ∗∗∗*P* = 0.0002 vs. sham, ##*P* = 0.0014 vs Veh (IL6), ∗∗*P* = 0.007 vs sham, #*P* = 0.046 vs Veh (IL1β), by 1-way ANOVA and Tukey post hoc tests. (J) Representative images of dihydroethidium (DHE) staining. (K) The 8-isoprostane concentrations are shown. Results are presented as mean ± SEM (n = 5). ∗∗∗∗*P* < 0.0001 vs sham, ##*P* = 0.0054 vs Veh, by 1-way ANOVA and Tukey post hoc tests. FU = fluorescence; INF = infarction; I/R = ischemia reperfusion; other abbreviations as in Figure 1.

Figure 2A shows the representative photographs of the heart sections stained with Evans blue dye to delineate the AAR and 2,3,5-triphenyl tetrazolium chloride to delineate the infarct area at 24 hours after reperfusion. Administration of MRC-Q significantly reduced the ratios of infarct area to AAR and of infarct area to LV, respectively (Figure 2B). There were no significant differences in the AAR/LV ratios between the 2 groups. Furthermore, treatment with MRC-Q significantly increased LV ejection fraction and fractional shortening in mice at 24 hours after myocardial I/R as measured by echocardiography (Figures 2C and 2D). Treated mice with MRC-Q also reduced the plasma levels of troponin I and creative kinase isoenzyme MB, a marker of heart damage, at 24 hours after I/R (Figure 2E).

Apoptosis is the key feature of various pathological heart conditions. Histological analysis revealed that apoptotic cells induced by I/R injury were less observed in the MRC-Q treatment group (Figures 2F and 2G). Caspase3/7 is one of the key molecules in apoptosis signaling. Of note, Figure 2H demonstrated that MRC-Q treatment attenuated caspase 3/7 production induced by myocardial I/R injury. Because inflammation contributes to myocardial injury after I/R, proinflammatory cytokines were also evaluated by quantitative reverse transcription–PCR in those animals. TNF-α, IL6, and IL1B, were reduced by MRC-Q treatment after I/R (Figure 2I). To investigate ROS production in the ischemic heart, we analyzed DHE staining by fluorescence microscopy at 24 hours after I/R injury with or without MRC-Q. Ischemic injury increased ROS production in the heart. However, this induction was attenuated by MRC-Q treatment (Figure 2J). In addition, 8-isoprostane production was less detected in the MRC-Q treatment group compared with the vehicle treatment group (Figure 2K).

### Mode of uptake of MRC-Q into H9c2 cells and its antiapoptotic effects

**Figure 3:**
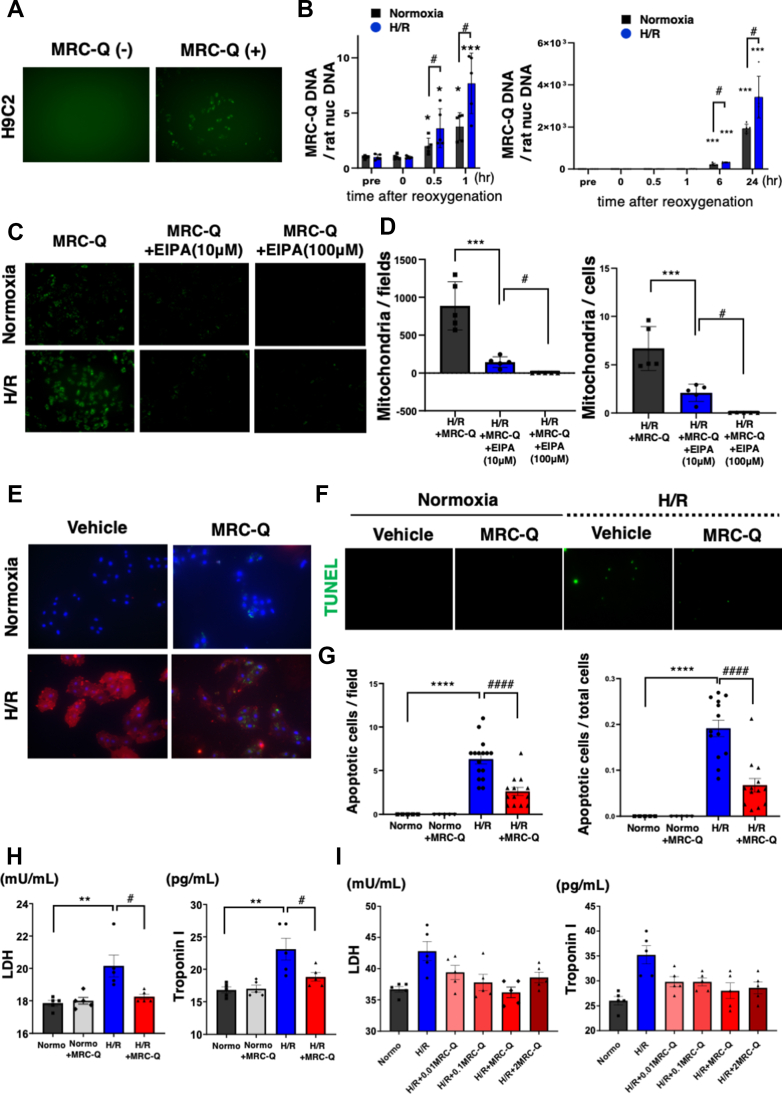
MRC-Q Decreases Apoptotic Cells After I/R Injury (A) Results from immunohistochemistry of H9c2 cells showing MRC-Q in green fluorescence. (B) Time-dependent changes in MRC-Q uptake. MRC-Q mRNA in H9c2 cells of normoxia (normo) and hypoxia (12-hour) group were quantified by polymerase chain reaction. Results are presented as mean ± SEM (n = 5). ∗*P* < 0.05, ∗∗∗*P* < 0.001 vs 0 hours in same group, #P < 0.05 vs normoxia group. (C,D) Results from immunohistochemistry of H9c2 cells showing MRC-Q in green fluorescence. Promotion of MRC-Q uptake by hypoxia and inhibition of MRC-Q uptake by ethylisopropylamiloride (EIPA). The quantitative statistical chart of MRC-Q in H9c2 cells with treated with EIPA. Results are presented as mean ± SEM (n = 5). ∗∗∗*P* = 0.0001 vs hypoxia/reoxygenation (H/R)+MRC-Q, #*P* = 0.047 vs H/R+MRC-Q EIPA (10 μ mol/L) (C), ∗∗∗*P* = 0.0007 vs H/R+MRC-Q, #*P* = 0.041 vs H/R+MRC-Q EIPA (10 μmol/L) (D). (E) Annexin V staining on H9c2 cells showing apoptotic cardiomyocytes in red fluorescence, MRC-Q in green fluorescence, and blue fluorescence (staining nucleus [nuc]). (F) Apoptosis detected by terminal uridine end-nick labeling (TUNEL) staining. (G) The quantitative statistical chart of apoptotic nuclei in H9c2 cells with treated MRC-Q. Results are presented as mean ± SEM. ∗∗∗∗*P* < 0.0001 vs normoxia, ####*P* < 0.0001 vs H/R, by 1-way ANOVA and Tukey post hoc tests. (H) Effects of MRC-Q on H/R induced H9c2 cell injury. Leakage of lactate dehydrogenase (LDH) and concentrations of troponin I (TnI). Results are presented as mean ± SEM. ∗∗*P* = 0.0024 vs normoxia, #*P* = 0.011 vs H/R (LDH), ∗∗*P* = 0.0016 vs normoxia, #*P* = 0.030 vs H/R (TnI), by 1-way ANOVA and Tukey post hoc tests. (I) Differential effects of MRC-Q concentration on H9c2 cell injury induced by H/R. LDH leakage and TnI concentrations. Results are presented as mean ± SEM (n = 5). Abbreviations as in Figures 1 and 2.

Next, purified green fluorescent protein–labeled human umbilical vein endothelial cell–derived mitochondria were added to a rat cardiomyocyte cell line and were observed to be taken up into cardiomyocytes (Figure 3A), which was also confirmed by quantitative PCR detecting human mitochondrial DNA in rat cardiomyocytes in a time-dependent manner (Figure 3B). However, human mitochondrial DNA could not be detected 7 days after MRC-Q treatment (Supplemental Figure 2A). The short-term duration of this intracellular presence of MRC-Q was supported by the results that it is no longer detectable in the heart at 28 days after MRC-Q administration (Supplemental Figure 2B). Interestingly, when the myocytes were exposed to H/R, a greater number of mitochondria were incorporated into the myocytes compared to normal nonischemic conditions (Figure 3B). In addition, we confirmed that cell viability was improved in the mitochondria-treated group 96 hours after H/R (data not shown). Furthermore, in vitro studies revealed that MRC-Q uptake was inhibited by ethylisopropylamiloride in a dose-dependent manner (Figures 3C and 3D). Also, mitochondrial DNA of human (ie, MRC-Q) origin detected in cardiomyocytes was inhibited by ethylisopropylamiloride treatment (Supplemental Figure 2C). These data indicated at least in part of the mitochondrial uptake mechanism in cardiomyocytes is mediated through macropinocytosis. To examine the intracellular effects of mitochondria after uptake, we examined the effects on apoptosis induced by H/R. Results demonstrated that MRC-Q–treated cells showed less apoptosis detected by both annexin V staining (Figure 3E), an indicator of early apoptosis, and TUNEL staining, an indicator of late apoptosis (Figures 3F and 3G). Furthermore, Figure 3H demonstrated that MRC-Q treatment attenuated cardiac enzymes (ie, lactate dehydrogenase and troponin I) in the cultured medium under H/R injury condition. These effects were observed in the dose-dependent manner of MRC-Q (Figure 3I).

### Potential mechanism of MRC-Q treatment on an antiapoptotic effect against I/R injury

**Figure 4:**
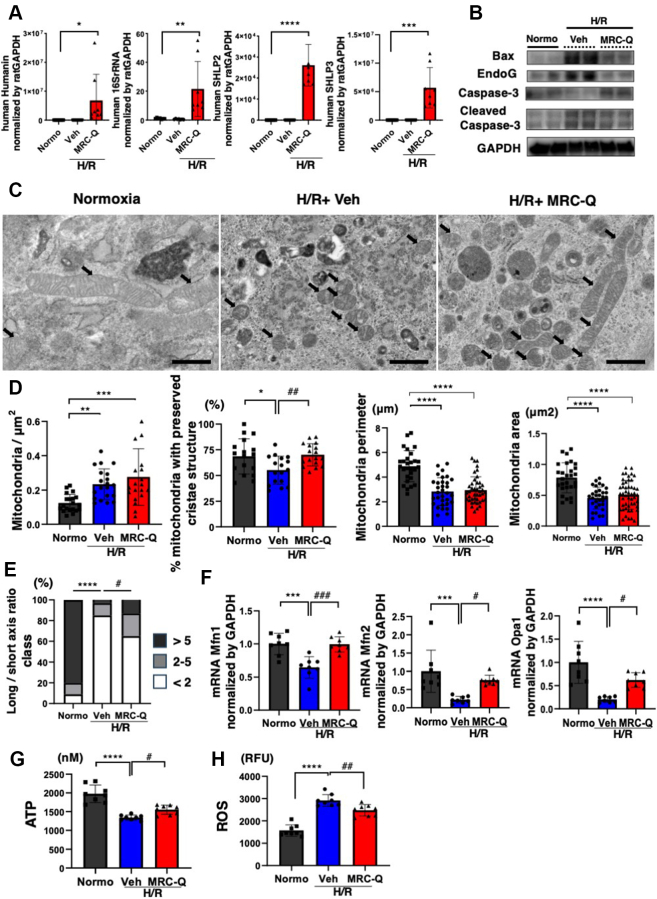
MRC-Q Preserves Mitochondrial Function In Vitro (A) The mRNA expression levels of humanin, 16SrRNA, and small humanin-like peptides 2 and 3 (SHLP2 and SHLP3). Results are presented as mean ± SEM (n = 5). ∗*P* = 0.047 vs normoxia in humanin, ∗∗*P* = 0.0074 vs normoxia in 16SrRNA, ∗∗∗∗*P* < 0.0001 vs normoxia in SHLP2, ∗∗∗*P* = 0.0001 vs normoxia in SHLP3, by 1-way ANOVA and Tukey post hoc tests. (B) Representative photographs by Western blot of Bax, caspase 3, cleaved caspase 3, and GAPDH. (C) Representative electron microscopy images of mitochondria from different groups of H9c2 cells following 12 hours of hypoxia and 24 hours of reperfusion. Black arrows indicate mitochondria. Bar = 1.0 μm. (D) Number of mitochondria per micrometers squared, percentage of mitochondria with preserved cristae structure, mitochondria perimeter, and area measurements in each field of view. Results are presented as mean ± SEM (n = 20). ∗∗*P* = 0.008, ∗∗∗*P* = 0.0002 vs normoxia in number of mitochondria per micrometers squared, ∗*P* = 0.021, ##*P* = 0.0091 vs Veh in percentage of mitochondria with preserved cristae structure, ∗∗∗∗*P* < 0.0001 vs normoxia in mitochondria perimeter and area measurements, by 1-way ANOVA and Tukey post hoc tests. (E) Percentage of mitochondria with long-/short-axis ratio class in each field of view. ∗∗∗∗*P* < 0.0001 vs normoxia, #*P* = 0.035 vs Veh, by contingency table analysis: Chi-square tests. (F) The mRNA expression levels of Mfn1, Mfn2, and Opa1. Results are presented as mean ± SEM (n = 5). ∗∗∗*P* = 0.0003 vs normoxia, ###*P* = 0.0003 vs Veh in Mfn1, ∗∗∗*P* = 0.0006 vs normoxia, #*P* = 0.014 vs Veh in Mfn2, ∗∗∗∗*P* < 0.0001 vs normoxia, #*P* = 0.017 vs Veh in Opa1, by 1-way ANOVA and Tukey post hoc tests. (G) Changes of intracellular adenosine triphosphate (ATP) concentration and (H) reactive oxygen species (ROS) activity in H9c2 cells. Results are presented as mean ± SEM (n = 5). ∗∗∗∗*P* < 0.0001 vs normoxia, #*P* = 0.035 vs vehicle in ATP, ∗∗∗∗*P* < 0.0001 vs normoxia, #*P* = 0.007 vs vehicle in ROS, by 1-way ANOVA and Tukey post hoc tests. RFU = rate fluorescence; other abbreviations as in Figures 1, 2, and 3.

To further investigate the beneficial mechanisms of MRC-Q after I/R injury, we next focused on the mitochondrial-derived peptide (MDP) because previous research demonstrated that the peptides humanin and small humanin-like peptides (SHLP) 2 and 3 from mitochondria induced antiapoptotic signaling. Figure 4A demonstrated that human cell–derived MDPs were detected in rat cells, indicating that MRC-Q potentially transferred MDPs to the injured myocytes against H/R injury. In fact, apoptotic signaling was attenuated by MRC-Q treatment after H/R injury (Figure 4B). Electron microscopy revealed that the number of mitochondria with preserved cristae structure was decreased under H/R condition; however, MRC-Q treatment protects this type of damage (Figures 4C and 4D). In addition, long mitochondria classified by long- and short-axis ratio were recovered by MRC-Q treatment against H/R injury (Figure 4E, Supplemental Figures 4A to 4C). These results were supported by the evidence that expressions of mitochondria quality control molecules in terms of Mfn1, Mfn2, and Opa1 were recovered by MRC-Q treatment after H/R injury (Figure 4F). The same effect of MRC-Q administration on the promotion of Mfn1, Mfn2, and OPA1 expression under ischemic conditions was suggested in the in vivo model (Supplemental Figures 3A to 3C). As a result, more ATP production and less ROS production were detected in the MRC-Q group after H/R injury (Figures 4G and 4H).

### MRC-Q treatment prevents fibrotic change and LV remodeling in the setting of heart failure after I/R

**Figure 5:**
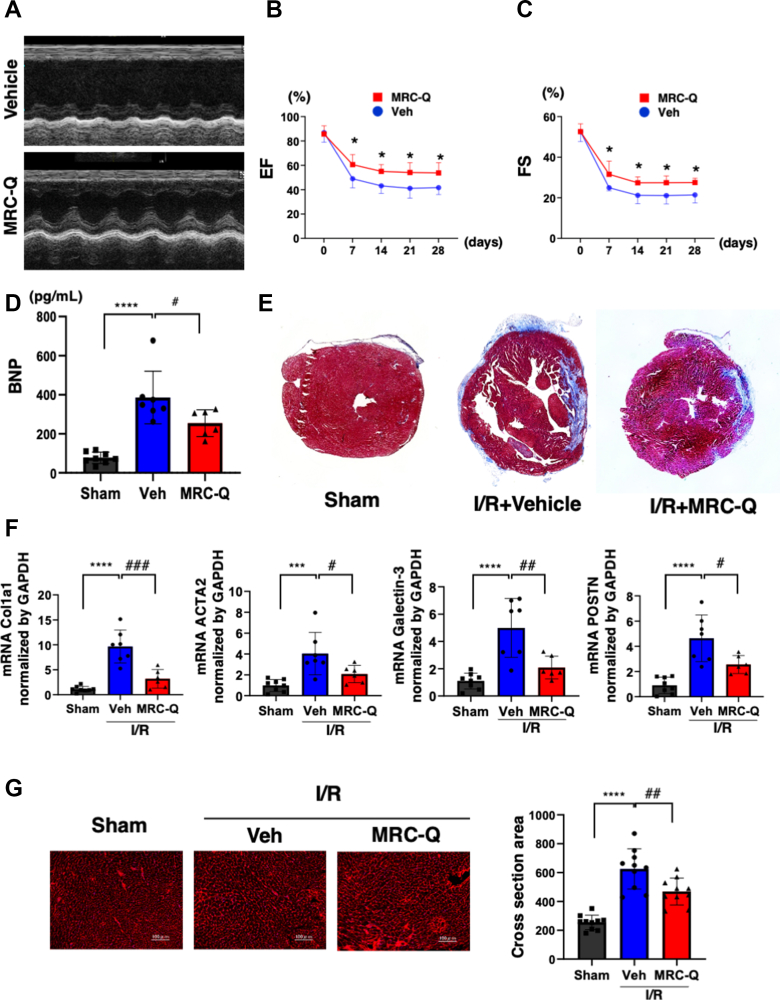
MRC-Q Improves Cardiac Function and LV Remodeling for 4 Weeks (A) Representative echocardiograms images from mice hearts under I/R injury after 4 weeks (Veh and with MRC-Q treatment). (B) EF and (C) FS percentages were examined by echocardiography. Results are presented as mean ± SEM from Veh (n = 7) and MRC-Q (n = 6). ∗*P* < 0.05 vs Veh, by 2-way ANOVA and Tukey post hoc tests. (D) Plasma B-type natriuretic peptide (BNP) concentration 4 weeks after I/R injury. Results are presented as mean ± SEM from sham (n = 8), Veh (n = 7), and MRC-Q (n = 6). ∗∗∗∗*P* < 0.0001 vs sham, #*P* = 0.035 vs Vehi, by 1-way ANOVA and Tukey post hoc tests. (E) Representative images of heart sections stained with Masson trichrome are shown: The fibrotic scar appears blue, and the viable myocardium is red. (F) The mRNA expression levels of Col1a1, ACTA2, galectin 3, and POSTN. Results are presented as mean ± SEM from sham (n = 8), Veh (n = 7), and MRC-Q (n = 6). ∗∗∗∗*P* < 0.0001 vs sham, ###*P* = 0.0001 vs Veh in Col1a1, ∗∗∗*P* = 0.0007 vs sham, #*P* = 0.036 vs Veh in ACTA2, ∗∗∗∗*P* < 0.0001 vs sham, ##*P* = 0.003 vs Veh in galectin 3, ∗∗∗∗*P* < 0.0001 vs sham, #P = 0.016 vs Veh in POSTN, by 1-way ANOVA and Tukey post hoc tests. (G) Representative images of heart sections stained with wheat germ agglutin are shown. Results are presented as mean ± SEM. ∗∗∗∗*P* < 0.0001 vs sham, ##*P* = 0.004 vs Veh, by 1-way ANOVA and Tukey post hoc tests. Abbreviations as in Figures 1 and 2.

Next, we investigated whether cardioprotective effect was sustained not only in acute phase but also in chronic phase, resulted by the inhibition of LV remodeling and reduction of heart load. Results showed that MRC-Q administration during reperfusion was effective in preserving cardiac function for at least 4 weeks (Figures 5A to 5C) and improved blood B-type natriuretic peptide levels (Figure 5D), a biomarker of heart failure. Histological evaluation showed reduced fibrosis and wall thinning around the infarcted nest in the chronic phase of postoperative day 28 (Figure 5E). Fibrosis-promoting markers in the myocardium during the chronic phase were reduced in the MRC-Q group (Figure 5F). It was also observed histologically that myocyte hypertrophy associated with LV remodeling after myocardial infarction was reduced by MRC-Q administration (Figure 5G). These results suggest that the acute cardioprotective effect of MRC-Q during reperfusion could lead to prevention of LV remodeling and heart failure during the chronic phase.

### Cardioprotective effect of MRC-Q treatment on myocardial I/R injury in a preclinical pig model

**Figure 6:**
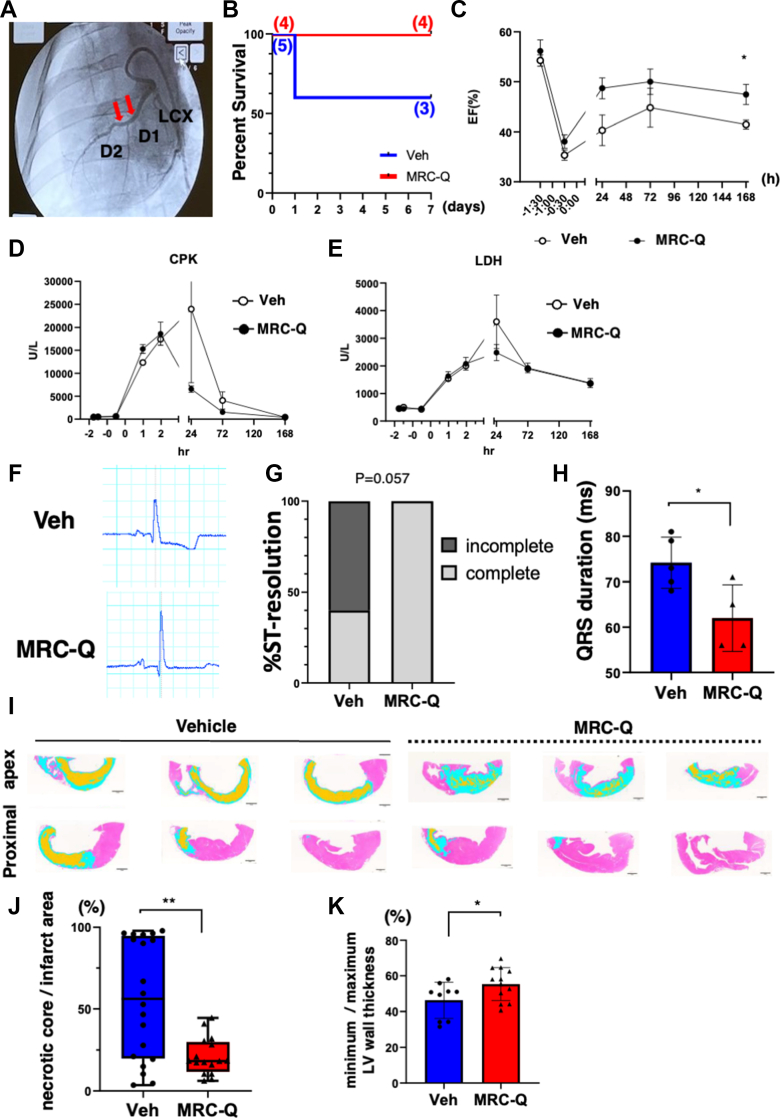
Effect of MRC-Q in Pig With 90 Minutes I/R Injury (A) Illustration of the pig left coronary artery anatomy before balloon inflation. A balloon was inflated just above the first diagonal (D1) branch. (B) Survival rate after myocardial I/R injury. (C) EF percentage was examined by echocardiography. Results are presented as mean ± SEM from Veh (n = 5) and MRC-Q (n = 4). ∗*P* < 0.05 vs vehicle, by 2-way ANOVA and Tukey post hoc tests. (D) Circulating creatine phosphokinase levels and (E) circulating LDH levels. (F-H) Representative electrogram recordings from a pig in 2 hours after I/R operation. Duration of indicated intervals is given in milliseconds. Veh group showed incomplete resolution and prolongation of the QRS duration after I/R injury. Results are presented as mean ± SEM from Veh (n = 5) and MRC-Q (n = 4). ∗*P* = 0.025 vs Veh. (I) Representative images of histological examination of infarct area (blue) and necrosis (orange) in heart 7 days after 90 minutes of I/R injury. (J) Comparison of necrotic/infarct percentage of pig hearts’ I/R injury. Data are shown as median (IQR) and analyzed by Mann-Whitney *U* test from Veh (n = 20) and MRC-Q (n = 16), 4 heart sections per pigs. ∗∗*P* = 0.006 vs Veh. (K) Comparison of minimum/maximum LV wall percentage of pig hearts’ I/R injury. Results are presented as mean ± SEM from Veh (n = 3) and MRC-Q (n = 4), 3 points per pigs. ∗*P* = 0.045 vs Veh. LCX = left circumflex [artery]; U/L = units/L; other abbreviations as in Figures 1, 2, and 3.

Finally, we examine the effects of MRC-Q on cardiac injury in a large-animal model of I/R, female pigs were subjected to 90 minutes of ischemia of the LAD artery following reperfusion. Intracoronary injection of MRC-Q or vehicle was performed via the wire lumen of the catheter just before the reperfusion (Figure 6A). The MRC-Q group did not promote adverse events such as induction of arrhythmias (data not shown) or worsening mortality compared to the vehicle control group (Figure 6B). In the control group, 2 of the 5 animals died of sudden death associated with pulseless electrical activity or ventricular fibrillation, but no deaths were observed in the treatment group (Figure 6B). However, the treatment group showed consistent improvement in cardiac function over 7 days (Figure 6C). Measurement of myocardial enzyme (in terms of creatine phosphokinase, lactate dehydrogenase) in the circulating blood showed that leakage of myocardial enzymes were reduced in the treatment group compared to the control group at 24 hours (Figures 6D and 6E). Evaluation of the electrocardiographic waveforms in terms of ST-segment resolution revealed 60% of animals in the control group had incomplete ST-segment resolution, whereas complete ST-segment resolution was achieved in all animals in the MRC-Q group (Figures 6F and 6G). Increased QRS duration reflects LV myocardial dysfunction, but the MRC-Q group had less increased QRS duration after treatment compared to the control group (Figure 6H). Histological examination revealed a reduction in the infarct size and LV remodeling findings such as wall thinning in the MRC-Q group compared to the control group (Figures 6I to 6K).

## Discussion

Our study provides the first evidence that: 1) intact mitochondria formula MRC-Q isolated by a novel technique can be preserved and ATP production could be maintained; 2) MRC-Q administration during reperfusion can reduce cardiac I/R injury; 3) MRC-Q is taken up into cardiomyocytes by macropinocytosis and secretes MDPs, thereby preventing cell injury by reducing endogenous proapoptotic signals; 4) MRC-Q promotes endogenous mitochondrial dynamics contributing to the mitochondrial quality control in injured myocytes; 5) preclinical studies in large animals have also confirmed the cardioprotective effect of MRC-Q administration against I/R injury (Central Illustration).

**Central Illustration:**
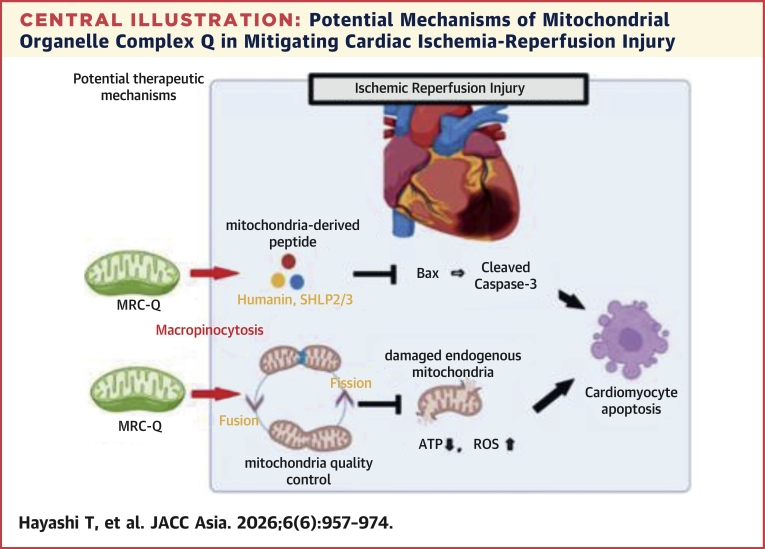
Potential Mechanisms of Mitochondrial Organelle Complex Q in Mitigating Cardiac Ischemia-Reperfusion Injury The administration of mitochondrial organelle complex Q (MRC-Q) during reperfusion is hypothesized to attenuate cardiac ischemia-reperfusion injury through multiple mechanisms. MRC-Q is internalized by cardiomyocytes, at least partially via macropinocytosis, and releases mitochondrial-derived peptides. These peptides contribute to cellular protection by suppressing endogenous proapoptotic signaling pathways. Furthermore, MRC-Q enhances endogenous mitochondrial dynamics, thereby supporting mitochondrial quality control. This process is associated with increased adenosine triphosphate (ATP) production and reduced reactive oxygen species (ROS) generation in injured myocytes, collectively promoting myocardial recovery. SHLP2/3 = small humanin-like peptides 2 and 3.

Ischemic heart disease, including myocardial infarction, is the leading cause of death worldwide.^1^ It is also known that endogenous mitochondria are damaged during myocardial infarction and I/R, and that this is an important pathogenic mechanism leading to impaired cardiac function and acute complications.^7^ Therefore, researchers around the world have been intensively studying the quantitative and qualitative improvement of damaged endogenous mitochondria by promoting biogenesis and quality control, or developing cardioprotective therapies targeting their downstream signaling.^41^^,^^42^

The therapeutic strategy we are testing and advocating here is an innovative and bold novel approach to organ protection by directly replenishing mitochondria themselves into injured organs.

Although there have been few reports of the development of mitochondrial replacement therapy from a limited number of institutions, all previous reports were based on autologous mitochondria and were limited to the therapeutic strategy of administration immediately after isolation because the preservation potential of isolated mitochondria is unknown.^32^^,^^43^ In other words, the conventional method of administration at the time of reperfusion is not realistic for unexpected sudden-onset diseases, including acute coronary syndromes, taking into account the time required for the isolation of autologous mitochondria.

The MRC-Q extracted by the method we have discovered this time can be stored and commercialized. This means that it can be used immediately at any time, making mitochondrial replacement therapy feasible: for example, for emergency diseases with sudden onset, for which early intervention is extremely important.

Mitochondria change in number and shape within the cell to regulate quality and function. They are among the important organelles that not only produce energy internally but also have multifaceted functions including cell death signaling.^44^

Recent reports indicate that mitochondria secrete MDP, which exerts protective effects against atherosclerosis, aging, glucose intolerance, and lipid abnormalities through intracellular signal transduction, such as antiapoptotic and anti-inflammatory effects.^45^ For instance, recent reports have shown that the mitochondrial-derived peptide S14G-humanin, an analogue of humanin, reduced infarct size in a pig model of 60-minute ischemia and following reperfusion.^46^ In addition, mitochondria are also known to act cytoprotectively via intercellular signal transduction, because they are actively transferred between cells even in the body.^47^^,^^48^

In the present study, we have confirmed that exogenously administered mitochondria are taken up into injured cardiomyocytes and potentially secrete MDPs such as humanin, SHLP2, and SHLP3, which would be among the potential mechanisms for cardioprotection. Furthermore, we found that mitochondria contribute to antiapoptosis of cardiomyocytes by suppressing the up-regulation of Bax, caspase 3, and other factors that promote apoptosis under I/R conditions. At the same time, it was also suggested that the therapy may activate endogenous mitochondrial dynamics, which may lead to the up-regulation of ATP production and reduction of ROS.^49^

In this study, we evaluated not only the acute-phase effects but also the chronic-phase cardiac function, revealing an intriguing observation: human mitochondrial DNA becomes undetectable in cardiomyocytes by day 7 (Supplemental Figure 2A), yet functional benefits persist at 4 weeks (Figures 5B and 5C). Two potential mechanisms are proposed to underlie these findings.

The first mechanism posits that exogenously administered MRC-Q exerts its effects during the acute phase of I/R injury (within 24 hours post onset) by reducing ROS production and suppressing apoptosis. This leads to a reduction in the infarcted myocardial area (Figure 2), which in turn contributes to the suppression of LV remodeling and the preservation of cardiac function in the chronic phase (Figure 5). These effects are analogous to those observed with early reperfusion therapy via percutaneous coronary intervention.

The second mechanism suggests that exogenously administered MRC-Q enhances the function of “endogenous” mitochondria, particularly through improved mitochondrial dynamics and quality control (Figure 4). This improvement may account for the sustained cardiac function benefits observed even after MRC-Q itself is cleared from the system.

Finally, we also conducted a preclinical study in pigs to further test efficacy and safety. We demonstrated that exogenous mitochondria administered intracoronary simultaneously with reperfusion improved acute cardiac function for at least 1 week after administration, leading to a reduction in infarct size and prevention of wall thinning.

Furthermore, the study confirmed the absence of adverse events such as microembolization (onset or prolongation of slow flow/no-reflow), induction of arrhythmias, and increased mortality in the treatment group compared to the control group (safety study). These results suggest that this treatment may soon be feasible in clinical practice.

### Study limitations

First, the sample size of each group is limited in the large animal model experiment conducted this time using pigs, which limits detailed examination and evaluation. Second, there are insufficient data to determine whether MDP derived from MRC-Q itself directly suppresses apoptosis-inducing signals in our model. A future study to overcome these limitations would be warranted.

## Conclusions

Our findings offer insights into the elucidation of the therapeutic potential of MRC-Q treatment against myocardial I/R injury.

### Data and Materials Availability

All data are available in the main text or the Supplemental Appendix.

## Funding Support and Author Disclosures

This work was partially supported by grants 22K08201 (to Y.S.) from the Ministry of Education, Culture, Sports, Science and Technology of Japan. The authors have reported that they have no relationships relevant to the contents of this paper to disclose.
