# Supplementary material for: Novel Allogeneic Mitochondria and Associated Organelle Complex Treatment Prevents Myocardial Ischemia-Reperfusion Injury Through Anti-Apoptotic Effects
Source: JACC Asia. 2025 Nov 18;6(6):957–74. doi: 10.1016/j.jacasi.2025.09.022 (PMC13244106; doi:10.1016/j.jacasi.2025.09.022)
Supplement: Supplementary Material [file mmc1.docx]

**Supplemental Figure 1.** (**A**) ECG waveforms: normal Heart (left) and ischemic heart (right).

**Supplemental Figure 2.** Human mitochondrial DNA detected in (**A**) H9C2 cells 7days after treatment, (**B**) ischemic heart 28 days after treatment. (**C**) Human mitochondrial DNA detection was blocked by EIPA (micropinocytosis inhibitor).

**Supplemental Figure 3. Mitofusion signaling were upregulated by MRC-Q treatment in a murine I/R model.** (**A**) (**B)** (**C**) The mRNA expression levels of Mfn1, Mfn2, and OPA1. Results are presented as mean ± SEM from sham (n=5), Veh (n=5) and MRC-Q (n=5). ****P<0.0001 vs. sham, #P<0.05, ###P < 0.001 vs Vehicle, by 1-way ANOVA and Tukey post hoc tests.

**Supplemental Figure 4. Electron microscopy.** Representative images of H9C2 cells under normoxia or hypoxia followed by reoxygenation with or without MRC-Q. These images were acquired at a magnification of ×10,000. (A) Perinuclear region. or (B) Remote nuclear region. (C) Representative images obtained at a lower magnification of ×3,000.

Primer Sequence

| *mouse TNF-α(F)* | AGCCCCCAGTCTGTATCCTT |
| --- | --- |
| *mouse TNF-α(R)* | CTCCCTTTGCAGAACTCAGG |
| *mouse IL-6 (F)* | AGTTGCCTTCTTGGGACTG |
| *mouse IL-6 (R)* | TCCACGATTTCCCAGAGAAC |
| *mouse IL-1β(F)* | TGACAGTGATGAGAATGACCTGTTC |
| *mouse IL-1β(R)* | TTGGAAGCAGCCCTTCATCT |
| *rat GAPDH (F)* | ATGACTCTACCCACGGCAAG |
| *rat GAPDH (R)* | CTGGAAGATGGTGATGGGTT |
|  |  |
| *human mt DNA-1 (F)* | AGACGGGTGAGACAGCTGCACCTTTTC |
| *human mt DNA-1 (R)* | CGAGAGCATCAAGTGCAGGCATTAGAG |
| *rat nuc DNA (F)* | GGTGTACTTGAGCAGAGCGCTATAAAT |
| *rat nuc DNA (R)* | CACTTACCCACGGCAGCTCTCTAC |
|  |  |
| *human HUMANIN (F)* | TGTCAACCCAACACAGGCATG |
| *human HUMAIN (R)* | AAACAGGCGGGGTAAGATTTG |
| *human 16sRNA (F)* | CACTGTCAACCCAACACAGG |
| *human 16sRNA (R)* | GGCAGGTCAATTTCACTGGT |
| *human SHLP2 (F)* | TGGTGATAGCTGGTTGTCCA |
| *human SHLP2 (R)* | AGGCTTATGCGGAGGAGAAT |
| *human SHLP3 (F)* | ATTGAAACCTGGCGCAATAG |
| *human SHLP3 (R)* | TGGACAACCAGCTATCACCA |
|  |  |
| *rat Tfam (F)* | ATCAAGACTGTGCGTGCATC |
| *rat Tfam (R)* | AGAACTTCACAAACCCGCAC |
| *rat Mfn1 (F)* | CGCCTGTCTGTTTTGGTTGA |
| *rat Mfn1 (R)* | GCATTGACTTCACTGGTGCA |
| *rat Mfn2 (F)* | AGTCGGTTGGAAGTCACTGT |
| *rat Mfn2 (R)* | TGTACTCGGGCTGAAAGGAG |
| *rat Fis 1 (F)* | AAAGAGGAGCAGCGGGATTA |
| *rat Fis 1 (R)* | TGGGGCTCAGTCTGTAACAG |
| *rat Opa1 (F)* | CCGTGTGAGCAGAAGAACAC |
| *rat Opa1 (R)* | AGCCTCAAGGCCAACTATGT |
| *rat Drp1 (F)* | GCAGCCGTAGTCCCTCAAAGA |
| *rat Drp1 (R)* | CTCCACCTTTTGAAGCCAGG |
|  |  |
| *mouse Col1a1 (F)* | GAGCCCTCGCTTCCGTACTC |
| *mouse Col1a1 (R)* | TGTTCCCTACTCAGCCGTCTGT |
| *mouse Acta2 (F)* | TCAGCGCCTCCAGTTCCT |
| *mouse Acta2 (R)* | AAAAAAAACCACGAGTAACAAATCAA |
| *mouse Gal-3 (F)* | TTGAATCTGACCACTTCAAGGTT |
| *mouse Gal-3 (R)* | AGGTTCTTCATCCGATGGTTGT |
| *mouse Postn (F)* | TGCTGCCCTGGCTATATGAG |
| *mouse Postn (R)* | GTAGTGGCTCCCACAATGCC |
|  |  |
| *rat Endo G (F)* | TAGCGCGCTGGTCCTCCGGTA |
| *rat Endo G (R)* | GCTTGAGTGAGTCCTACTCCA |
| *rat Palmdelphin (F)* | CACACCCAGCAAAAGAGGAT |
| *rat Palmdelphin (R)* | AGGAGGCAGAGAATGAACGA |
|  |  |
| *mouse Mfn1 (F)* | GCAGACAGCACATGGAGAGA |
| *mouse Mfn1 (R)* | GATCCGATTCCGAGCTTCCG |
| *mouse Mfn2 (F)* | TGCACCGCCATATAGAGGAAG |
| *mouse Mfn2 (R)* | TCTGCAGTGAACTGGCAATG |
| *mouse Opa1 (F)* | ACCTTGCCAGTTTAGCTCCC |
| *mouse Opa1 (R)* | TTGGGACCTGCAGTGAAGAA |
